# Endothelial Twist1-PDGFB signaling mediates hypoxia-induced proliferation and migration of αSMA-positive cells

**DOI:** 10.1038/s41598-020-64298-5

**Published:** 2020-05-05

**Authors:** Akiko Mammoto, Kathryn Hendee, Megan Muyleart, Tadanori Mammoto

**Affiliations:** 1 Department of Pediatrics, Medical College of Wisconsin, Milwaukee, WI 53226 United States; 2 Department of Cell Biology, Neurobiology and Anatomy, Medical College of Wisconsin, Milwaukee, WI 53226 United States

**Keywords:** Growth factor signalling, Mechanisms of disease

## Abstract

Remodeling of distal pulmonary arterioles (PAs) associated with marked accumulation of pulmonary artery smooth muscle cells (PASMCs) represents one of the major pathologic features of pulmonary hypertension (PH). We have reported that the transcription factor Twist1 mediates hypoxia-induced PH. However, the mechanism by which endothelial Twist1 stimulates SMC accumulation to distal PAs in PH remains unclear. Here, we have demonstrated that Twist1 overexpression increases the expression of platelet-derived growth factor (PDGFB) in human pulmonary arterial endothelial (HPAE) cells. Hypoxia upregulates the levels of Twist1 and PDGFB in HPAE cells. When we implant hydrogel supplemented with endothelial cells (ECs) on the mouse lung, these ECs form vascular lumen structures and hypoxia upregulates PDGFB expression and stimulates accumulation of αSMA–positive cells in the gel, while knockdown of endothelial Twist1 suppresses the effects. The levels of Twist1 and PDGFB are higher in PAE cells isolated from idiopathic pulmonary arterial hypertension (IPAH) patients compared to those from healthy controls. IPAH patient-derived PAE cells stimulate accumulation of αSMA–positive cells in the implanted gel, while Twist1 knockdown in PAE cells inhibits the effects. Endothelial Twist1-PDGFB signaling plays a key role in αSMA–positive cell proliferation and migration in PH.

## Introduction

Pulmonary hypertension (PH) is a multifactorial life-threatening cardiopulmonary disorder^1,2^. It is characterized by a sustained increase in pulmonary arterial (PA) pressure, leading to right-sided heart failure^1,2^. The current therapeutic options only partially improve symptoms and survival^1,3^. Uncontrolled pulmonary artery smooth muscle cell (PASMC) proliferation and accumulation of PASMCs to normally non-muscularized distal PAs are the major histological characteristics of PH^2,4^. Thus, we need to understand the mechanism of abnormal SMC proliferation, migration, and their accumulation to distal PAs. In addition to lining vascular structures, endothelial cells (ECs) regulate various physiological functions by secreting angiocrine factors^5^. Deregulation of this paracrine mechanism leads to the development of diseases^5^. In PH pathology, abnormal ECs release factors that stimulate SMC proliferation (*e.g*., FGF-2^6^, serotonin^7^) or fail to produce factors that physiologically suppress SMC proliferation (*e.g*., apelin^8^), indicating that aberrant pulmonary arterial EC signaling plays key roles in abnormal SMC accumulation to distal PAs^2^.

The transcription factor Twist1 controls physiological and pathological angiogenesis in various organs including the lungs^9–14^. The levels of Twist1 are upregulated in the lungs of patients with pulmonary arterial hypertension (PAH; WHO group1 PH)^15,16^ and mice with type II bone morphogenetic protein receptor (Bmpr2) gene mutation^15^, a gene mutated in familial and idiopathic PAH^17^. Twist1 expression also increases in the patients with chronic lung diseases associated with PH such as pulmonary fibrosis^18,19^. We have demonstrated that endothelial Twist1 contributes to the pathogenesis of pulmonary fibrosis in a bleomycin-induced mouse pulmonary fibrosis model^12^. Twist1 is also involved in endothelial to mesenchymal transition (EndMT)^14,15,20^, which potentially contributes to the pathogenesis of PH^2,15,16,20–23^. We have reported that hypoxia-induced pathological accumulation of α-smooth muscle actin (αSMA)-positive cells, including SMCs and myofibroblasts, to distal PAs and increases in right ventricular systolic pressure (RVSP) are attenuated in Tie2-specific Twist1 conditional knockout (*Twist1*^*fl/fl*^*/Tie2-cre*) mouse lungs^20^. However, the mechanistic role of endothelial Twist1 in the vascular remodeling in PH has not been fully understood. Twist1 controls the expression of multiple angiogenic factors and receptors in ECs^12,13^ and exhibits crosstalk with other angiogenesis-related signaling pathways such as hypoxia-inducible factor-1α (HIF-1α)^24^, Wnt^25,26^, Notch^27^, phosphoinositide 3-kinase (PI3K)-AKT^28^, and transforming growth factor (TGF)-β^20,29^, which are involved in the pathogenesis of PH^1,2^. Twist1 also controls the expression of platelet-derived growth factor (PDGF)-related genes in tumor cells^30^. PDGFB controls SMC proliferation and migration, and excessive PDGFB expression contributes to PH pathology^1,31–33^.

Here we have demonstrated that endothelial Twist1 stimulates SMC DNA synthesis and migration by increasing PDGFB expression *in vitro* and mediates hypoxia-induced αSMA-positive cell accumulation in the gel implanted on the mouse lungs. Knockdown of endothelial Twist1 also inhibits accumulation of αSMA-positive cells in the gel supplemented with human IPAH patient-derived ECs and implanted on the mouse lungs. Endothelial Twist1-PDGFB signaling could therefore be one of the key pathways in the pathogenesis of PH.

## Materials and Methods

### Materials

Anti-Twist1, -PDGFB, -HIF-1α, and -αSMA antibodies were from Abcam (Cambridge, MA). HIF-1α antibody (Abcam; ab1) was validated in MCF7 (human breast adenocarcinoma cell line) cells treated with metformin hydrochloride, which decreases HIF1α expression, to decrease the levels of HIF1α in a dose dependent manner by immunocytochemistry (ICC). PDGFB antibody (Abcam; ab23914) was validated by detecting recombinant human PDGFBB protein. Anti-β-actin monoclonal antibody was from Sigma (St. Louis, MO). Anti-Twist1 antibody was from Santa Cruz Biotechnology (Dallas, TX). Staining with secondary antibody alone confirmed that there was no non-specific binding of the secondary antibody for immunohistochemistry (IHC) (Supplementary Fig. 3a). Recombinant PDGFB and PDGF blocking antibody were purchased from R&D (Minneapolis, MN). Human pulmonary arterial endothelial (HPAE) cells (Lonza) were cultured in EBM2 medium containing 5% FBS and growth factors (VEGF, bFGF and PDGF). Human pulmonary artery smooth muscle cells (HPASMCs) were purchased from Lonza and cultured in DMEM containing 5% FBS. De-identified human IPAH patient ECs were obtained from unused donor control lungs at time of transplantation via the Pulmonary Hypertension Breakthrough Initiative (PHBI) Network, which is funded by the Cardiovascular Medical Research and Education Fund (CMREF) and NIH-NHLBI. The study using these de-identified human cells has been determined and approved as Non-Human Subjects Research by the Medical College of Wisconsin Institutional Review Board (IRB PRO00029154). We obtained ECs isolated from PA (>5 mm in diameter) from females and males (4 control samples; 44.25 +/− 2.86 years old, 6 IPAH samples; 32.5 +/− 2.79 years old). The patient demographic information is in Table 1. These ECs were cultured in EBM2 medium containing 5% FBS and growth factors (VEGF, bFGF and PDGF).

**Table 1:** Sample demographics.

ID	Age	Sex	Race	Passage
Con-1	36	Female	White	3–5
Con-2	45	Female	White	4–6
Con-3	47	Male	White	4–6
Con-4	49	Female	White	4–6
PAH-1	27	Female	White	4–6
PAH-2	23	Female	White	3–5
PAH-3	32	Male	White	3–5
PAH-4	33	Female	White	3–5
PAH-5	40	Male	White	4–6
PAH-6	40	Female	White	3–5

### Plasmid construction and gene knockdown

Human Twist1 siRNA was previously described^20^. Lentiviral construct targeting human Twist1 (Twist1 shRNA,CCGGGCTGGACTCCAAGATGGCAAGCTCGAGCTTGCCATCTTGGAGTCCAGCTTTTT) was obtained from Sigma. The full-length Twist1 plasmid was from Addgene (Cambridge, MA) and pHAGE-Twist1-full was constructed as described^20^. As a control, plasmid with vector only was used. Generation of lentiviral vectors was accomplished by a five-plasmid transfection procedure as reported^34^. Viral supernatants were collected starting 48 h after transfection for four consecutive times every 12 h, pooled, and filtered through a 0.45 μm filter. Viral supernatants were then concentrated 100-fold by ultracentrifugation in a Beckman centrifuge for 1.5 h at 16500 rpm. HPAE cells were incubated with viral stocks in the presence of 5 μg/ml polybrene (Sigma) and 90–100% infection was achieved 3 days later^34^.

### Molecular biological and biochemical methods

Quantitative reverse transcription (qRT)-PCR was performed with the iScript reverse transcription and iTaq SYBR Green qPCR kit (BioRad, Hercules, CA) using the BioRad real time PCR system (BioRad). β2 microglobulin controlled for overall cDNA content as a reference gene. The primers used for human β2 microglobulin and Twist1 were previously described^12,13,20,34^. The primers used for human PDGFB were forward; 5′-CTCGATCCGCTCCTTTGATGA-3′ and reverse; 5′-CGTTGGTGCGGTCTATGAG-3′. The protein levels of human PDGFB in ECs were measured using ELISA (R&D systems) and normalized by the protein levels of total cell lysate. When we measured the PDGFB protein levels in the conditioned media (CM), we normalized the levels by the cell numbers.

### Mouse EC isolation

Mouse lung ECs were isolated from *B6-GFP* mouse lungs (Jackson Laboratories, stock # 004353, 2–3 week old) using anti-CD31 conjugated magnetic beads^20^. We cut mouse lung tissue from *B6-GFP* mouse into small pieces using small scissors and treated the tissue with 5 ml collagenase A (1 mg/ml) for 30 min at 37 °C. The tissue suspension was filtered through a 40 μm cell strainer (Falcon) to remove the undigested cell clumps and separate single cells. Cells were centrifuged (1000 rpm, 5 min) at room temperature (RT) and the pellet was resuspended into 0.5 ml RBC Lysis Buffer (Sigma, 1 min, RT). The lysis reaction was stopped by adding 10 ml 10% FBS/DMEM, centrifuged (1000 rpm, 5 min, RT), and the pellet was resuspended into 0.5 ml 4% FBS/PBS with APC anti-mouse CD31 (Biolegend, 1/100), incubated (20 min, on ice) and washed three times with 4% FBS/PBS. Cells were centrifuged (1000 rpm, 5 min, RT) and resuspended into 0.1 ml 4% FBS/PBS with anti-APC conjugated microbeads (Miltenyl Biotec, Somerville, MA), incubated (10 min, on ice) and washed three times with 4% FBS/PBS. The cells were then resuspended in 0.5 ml 4% FBS/PBS and CD31-positive ECs were magnetically separated using MACS column (Miltenyl Biotec) according to manufacturer’s instruction. To increase the purity of the magnetically separated fraction, the eluted fraction was enriched over a second new MACS column. Using this method, we obtained 5 × 10^5^ cells/mouse and FACS analysis confirmed that 82.6% of the cells are CD31+ and VE-cadherin+ cells (Supplementary Fig. 2a).

### *In vitro* hypoxia assay

At 80% confluence, HPAE cells were exposed to 1% O_2 _for 48 h in a hypoxia chamber (Billups-Rothenberg, Del Mar, CA). Cells were lysed for molecular and biochemical analysis. DNA synthesis of SMCs was analyzed by a BrdU incorporation assay. HPASMCs (DMEM with 2% serum) were treated with CM collected from HPAE cells with or without manipulation of Twist1 or in combination with PDGFB (10 ng/ml), pulsed with 5 μM BrdU for 2 h, immunostained and imaged using a confocal Leica SP5 microscope^20^. DNA synthesis of HPASMCs was also analyzed by measuring the number of BrdU^+^ cells using FACS (BD Biosciences BrdU flow kit)^35^. Since counting of BrdU^+^ cells and FACS analysis revealed similar trends (Supplementary Fig. 2b), we used counting of BrdU^+^ cells in this study. SMC migration was analyzed using a modified transwell migration assay^36^. The cells that migrated towards the conditioned media collected from HPAE cells with or without manipulation of Twist1 in 0.5% serum DMEM or supplemented with PDGFB (10 ng/ml) through the membrane were stained with Giemsa, counted and averaged in three independent experiments.

### Fibrin gel implantation on the mouse lung

The *in vivo* animal study was carried out in strict accordance with the recommendations in the Guide for the Care and Use of Laboratory Animals of the National Institutes of Health. The protocols were reviewed and approved by the Animal Care and Use Committee of Medical College of Wisconsin. Fibrin gel was fabricated as described^20,37^. Briefly, we added thrombin (2.5 U/ml) with angiogenic factors (VEGF and bFGF at 100 ng/ml) to the fibrinogen solution (12.5 mg/ml), mixed well, and supplemented the gel with HPAE cells labeled with GFP using lentiviral transduction or ECs isolated from *B6-GFP* mouse lungs (1 × 10^6^ cells). When we supplemented the gel with human ECs and implanted on nonobese diabetic/severe combined immunodeficiency gamma (NSG, Jackson Laboratories, stock # 005557) mouse lungs, commercially available human lung fibroblasts (ATCC, Old Town Manassas, VA, 2.5 × 10^5^ cells) were mixed in the gel to induce robust vascular network formation in the gel^38^. These fibroblasts were not labeled with GFP. We did not mix the gel with fibroblasts when we implanted gel supplemented with ECs isolated from *B6-mGFP* (background C57BL6) mouse lungs on syngenic *B6-mRFP *(Jackson Laboratories, stock # 005884) or C57BL6 (Jackson Laboratories, stock # 000664, 8–10 week old) mouse lungs, because similar vessel-like structures were formed without fibroblasts. Immune cells recruited from host C57BL6 mice may help forming vessel-like structures without supplementing fibroblasts by secreting angiogenic factors or stimulating other cells. Drops of the mixture were incubated at 37 °C for 30 min until they solidified. For gel implantation on the mouse lungs, NSG, *B6-mRFP*, or C57BL6 mice were mechanically ventilated and thoracotomy was performed in the fifth left intercostal space^12,37^. After thoracotomy, a small area of the left visceral pleura (0.5 mm^2^) was scraped using forceps and the fabricated fibrin gel was implanted on the mouse lung surface using fibrin glue. To evaluate the effects of hypoxia *in vivo*, NSG, *B6-mRFP* or C57BL6 mice, in which fibrin gel supplemented with HPAE cells or ECs isolated from *B6-GFP* mouse lungs was implanted, were housed in plexiglass chambers and exposed to 8.5 ± 0.5% O_2_ for 3 days^20^. For histological analysis, gels were fixed in 4% PFA overnight at 4 °C followed by OCT embedding and cryosectioning. Fluorescent images were taken on a Leica TCS SP5 confocal laser scanning microscope or a Zeiss LSM 510 confocal imaging system. Fluorescently labeled EC-derived vascular lumen structures and accumulation of αSMA-positive cells in the gel were evaluated in five different areas of the gel using ImageJ software^12,20,34,37^.

### Statistical analysis

All phenotypic analysis was performed by masked observers unaware of the identity of experimental groups. Error bars (SEM) and *p* values were determined from the results of three or more independent experiments. The F test (for two samples) or the Levene test (for more than two samples) was performed to confirm that the variances are homogeneous. Student’s t-test was used for statistical significance for two groups. For more than two groups, one-way ANOVA with a post-hoc analysis using the Bonferroni test was conducted.

## Results

### Endothelial Twist1 stimulates DNA synthesis and migration of SMCs through PDGFB *in vitro*

**Figure 1:**
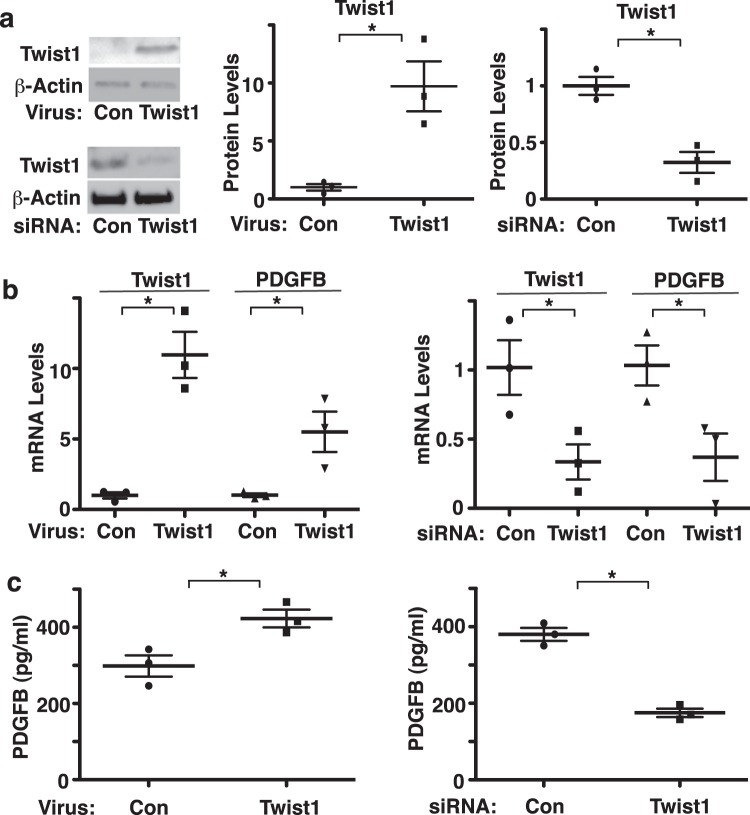
Twist1 induces PDGFB expression in HPAE cells *in vitro*. (**a**) Representative immunoblots showing Twist1 and β-actin protein levels in HPAE cells treated with lentivirus expressing Twist1 or control virus (vector alone; *left top*). Representative immunoblots showing Twist1 and β-actin protein levels in HPAE cells treated with Twist1 siRNA or control siRNA with irrelevant sequences (*left bottom*). Graphs showing the protein levels of Twist1 in HPAE cells treated with lentivirus expressing Twist1 or control virus (vector alone; *middle*) or Twist1 siRNA or control siRNA with irrelevant sequences (*right*; n = 3, *p < 0.05). Error bars represent s.e.m. (**b)** Graph showing the mRNA levels of Twist1 and PDGFB in HPAE cells treated with lentivirus expressing full-length Twist1 or control virus (*left*; n = 3, *p < 0.05). Graph showing the mRNA levels of Twist1 and PDGFB in HPAE cells treated with Twist1 siRNA or control siRNA with irrelevant sequences (*right*; n = 3, *p < 0.05). Error bars represent s.e.m. (**c**) Graph showing the protein levels of PDGFB in CM collected from HPAE cells treated with lentivirus expressing full-length Twist1 or control virus (*left*; n = 3, *p < 0.05). Graph showing the protein levels PDGFB in CM collected from HPAE cells treated with Twist1 siRNA or control siRNA with irrelevant sequences (*right*; n = 3, *p < 0.05). Error bars represent s.e.m.

**Figure 2:**
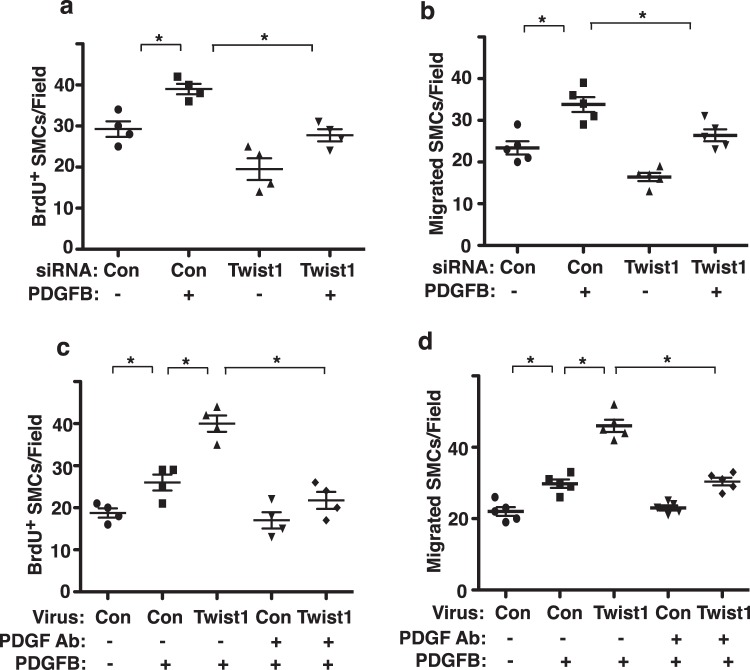
Endothelial Twist1 stimulates SMC DNA synthesis and migration through PDGFB *in vitro*. (**a**) Graph showing BrdU-positive HPASMCs treated with CM collected from HPAE cells treated with Twist1 siRNA or control siRNA with irrelevant sequences or in combination with PDGFB (n = 4, *p < 0.05). Error bars represent s.e.m. (**b**) Graph showing HPASMCs migrating towards CM collected from HPAE cells treated with Twist1 siRNA or control siRNA with irrelevant sequences or in combination with PDGFB (n = 5, *p < 0.05). Error bars represent s.e.m. (**c)** Graph showing BrdU-positive HPASMCs treated with CM collected from HPAE cells treated with lentivirus expressing Twist1 or in combination with PDGF inhibitory antibody or PDGFB (n = 4, *p < 0.05). As a control, CM was collected from HPAE cells treated with control virus (vector alone). Error bars represent s.e.m. (**d**) Graph showing HPASMCs migrating towards CM collected from HPAE cells treated with lentivirus expressing Twist1 or in combination with PDGF inhibitory antibody or PDGFB (n = 5, *p < 0.05). As a control, CM was collected from HPAE cells treated with control virus (vector alone). Error bars represent s.e.m.

The expression of Twist1 is upregulated in the lungs of patients with PAH^15,16^. Knockdown of endothelial Twist1 prevents hypoxia-induced increases in RVSP and accumulation of αSMA-positive cells to distal PAs in the mouse model^20^. However, the mechanism by which endothelial Twist1 controls SMC accumulation has not been fully understood. PDGFB is known to stimulate SMC proliferation and migration, and contributes to PH pathology^1,31–33^. Overexpression of Twist1 using lentiviral transduction (Fig. 1a, Supplementary Fig. 1) increased the mRNA and protein levels of PDGFB in HPAE cells by 5.2- and 1.3-times, respectively, compared to those treated with control virus (vector alone) as analyzed by qRT-PCR and ELISA, respectively (Fig. 1b,c). In contrast, Twist1 knockdown using siRNA transfection (Fig. 1a, Supplementary Fig. 1) decreased the mRNA and protein levels of PDGFB in HPAE cells, compared to those treated with control siRNA with irrelevant sequences (Fig. 1b,c). When we treated HPASMCs with CM collected from Twist1-knocked down HPAE cells in combination with supplementation with PDGFB, HPASMC DNA synthesis and migration induced by CM and PDGFB, analyzed using a BrdU incorporation assay and a transwell migration assay, respectively, were inhibited by 29% and 16%, respectively (Fig. 2a,b). Overexpression of Twist1 in HPAE cells stimulated SMC DNA synthesis and migration by 1.5- and 1.4- times, respectively, while treatment with a PDGF neutralizing antibody suppressed the effects (Fig. 2c,d), suggesting that endothelial Twist1 is required for SMC DNA synthesis and migration through PDGFB.

**Figure 3:**
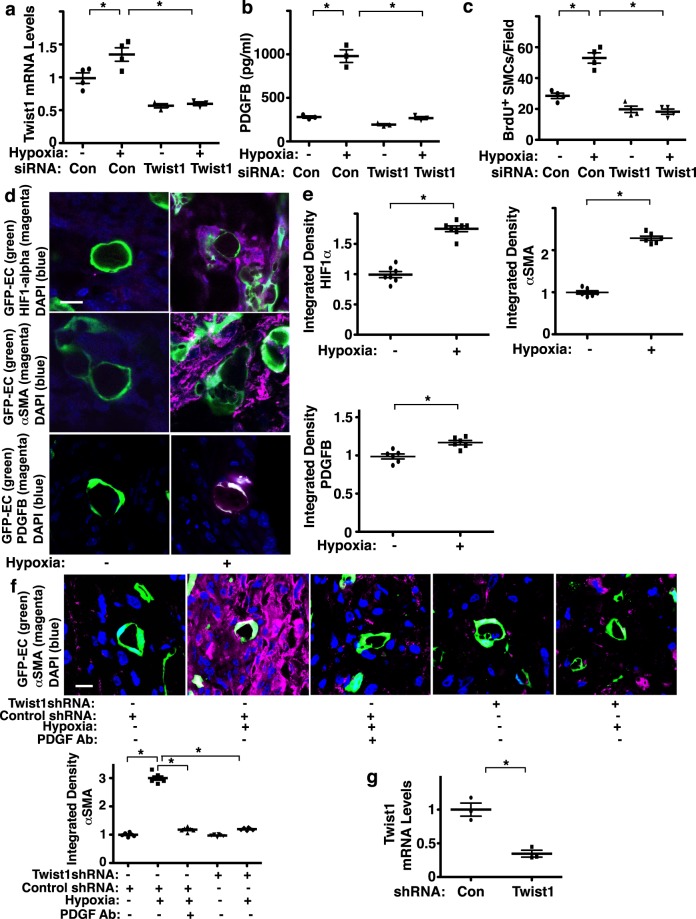
Endothelial Twist1 mediates hypoxia-induced DNA synthesis and accumulation of αSMA-positive cells in the gel implanted on the mouse lung through PDGFB. (**a**) Graph showing the mRNA levels of Twist1 in HPAE cells treated with hypoxia or in combination with control siRNA or Twist1 siRNA. As a control HPAE cells were treated with control siRNA with irrelevant sequences under normoxia (n = 4, *p < 0.05). Error bars represent s.e.m. (**b**) Graph showing the protein levels of PDGFB in CM collected from HPAE cells treated with hypoxia or in combination with control siRNA or Twist1 siRNA. As a control HPAE cells were treated with control siRNA with irrelevant sequences under normoxia (n = 3, *p < 0.05). (**c**) Graph showing BrdU-positive HPASMCs treated with CM collected from HPAE cells treated with hypoxia or in combination with control siRNA or Twist1 siRNA. As a control HPASMCs were treated with CM collected from HPAE cells treated with control siRNA with irrelevant sequences under normoxia (n = 4, *p < 0.05). Error bars represent s.e.m. (**d**) IF micrographs of fibrin gel supplemented with GFP-labeled *B6-GFP* mouse lung ECs implanted on C57BL6 mouse lung for 7 days and treated with hypoxia for the last 3 days; GFP-labeled blood vessel lumen structure, HIF1α expression and DAPI (*top*), GFP-labeled blood vessel lumen structure, αSMA expression and distribution and DAPI (*middle*), and GFP-labeled blood vessel lumen structure, PDGFB expression and distribution and DAPI (*bottom*) in the fibrin gel. Scale bar, 10 μm. (**e**) Graphs showing integrated fluorescent density of HIF1α (n = 7, *p < 0.05), αSMA(n = 6, *p < 0.05), and PDGFB (n = 6, *p < 0.05). Error bars represent s.e.m. (**f**) IF micrographs of fibrin gel supplemented with GFP-labeled HPAE cells treated with control shRNA or Twist1 shRNA, implanted on mouse lung for 7 days or in combination with exposure to hypoxia for the last 3 days and/or treatment with PDGF neutralizing antibody showing GFP-labeled blood vessel lumen structure, αSMA expression and distribution and DAPI in the fibrin gel. Scale bar, 10 μm. Graph showing the integrated fluorescent density of αSMA (n = 6, *p < 0.05). Error bars represent s.e.m. (**g**) Graph showing the mRNA levels of Twist1 in HPAE cells treated with lentivirus targeting Twist1 (Twist1 shRNA) or control virus (n = 3, *p < 0.05).

To explore more physiological relevance of endothelial Twist1 *in vitro*, we next examined the effects of hypoxia or in combination with manipulation of Twist1 expression on PDGFB expression in ECs. When we treated HPAE cells with hypoxia (1% O_2_) for 2 days, hypoxia increased the mRNA levels of Twist1 by 1.3 times and the protein levels of PDGFB measured by ELISA by 3.2 times, while Twist1 knockdown suppressed these effects (Fig. 3a,b). We also examined whether hypoxia treatment of HPAE cells stimulates SMC DNA synthesis. When we treated HPASMCs with CM collected from HPAE cells treated with hypoxia, BrdU incorporation was stimulated by 1.9-fold compared to those treated with CM of HPAE cells treated with normoxia (Fig. 3c). These effects were attenuated when HPASMCs were treated with CM collected from ECs treated with hypoxia in combination with Twist1 siRNA, suggesting that endothelial Twist1 mediates hypoxia-induced PDGFB expression and SMC DNA synthesis.

### Endothelial Twist1 mediates hypoxia-induced accumulation of αSMA-positive cells in the gel implanted on the mouse lungs

Blood vessels interact with other cellular and non-cellular components and build complex structures in an organ-specific manner^39–41^. Thus, to study vascular structures and cellular interactions in the lung, we developed a system to implant fibrin gel on the lung surface of living mice^12,20,37,41^. To study the effects of hypoxia on PDGFB expression and SMC accumulation in the lung, we implanted fibrin gel supplemented with ECs isolated from *B6-GFP* mouse lungs (background, C57BL6) on the C57BL6 mouse lung (8–10 week old) for 7 days and treated the mice with hypoxia (8.5%) for the last 3 days^12,37^. Confocal fluorescence images show that GFP-labeled ECs supplemented into the gel form a vascular lumen structure 7 days after implantation (Fig. 3d). Hypoxia treatment for the last 3 days stimulated HIF-1α expression, and induced PDGFB expression in ECs and accumulation of αSMA-positive cells in the gel (Fig. 3d,e). These αSMA-positive cells in the gel seem to be recruited from host mouse lungs; when we implanted fibrin gel supplemented with ECs isolated from *B6-GFP* mouse lungs to *B6-mRFP* mouse lungs and treated the mice with hypoxia for 3 days, αSMA-stained mRFP-positive cells accumulated in the gel (Supplementary Fig. 3b).

Consistent with previous report^20^ and the results using mouse lung ECs, when the gel was supplemented with HPAE cells, implanted on the mouse lung and treated with hypoxia for 3 days, hypoxia induced accumulation of αSMA-positive cells in the gel (Fig. 3f, Supplementary Fig. 3c). Consistent with previous report^20^, hypoxia also induced EndMT in the gel (Fig. 3f). Twist1 knockdown using lentivirus expressing Twist1 shRNA (Fig. 3g) inhibited the hypoxia-induced EndMT and accumulation of αSMA-positive cells in the gel (Fig. 3f). A PDGF neutralizing antibody also suppressed the hypoxia-induced accumulation of αSMA-positive cells in the gel (Fig. 3f). These results suggest that Twist1-PDGFB signaling mediates hypoxia-induced αSMA-positive cell migration and DNA synthesis in the lung.

### Twist1 controls PDGFB expression in IPAH patient PAECs

**Figure 4:**
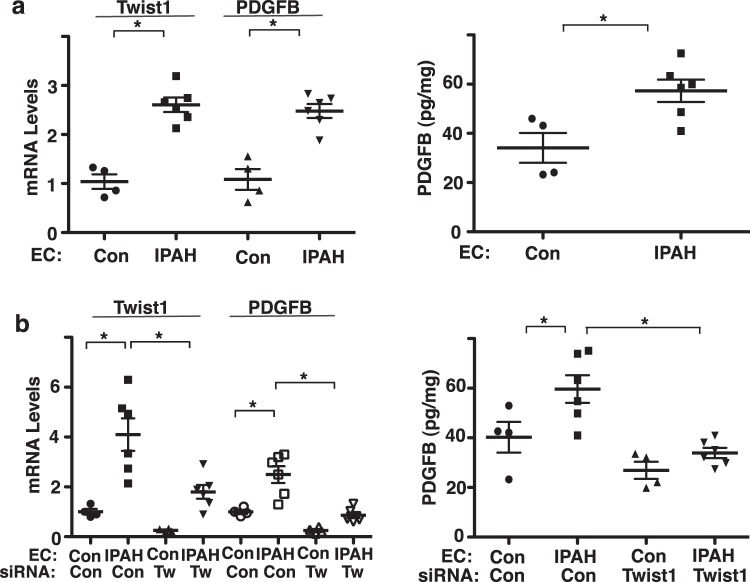
Twist1 mediates PDGFB expression in IPAH patient ECs *in vitro*. (**a**) Graph showing the mRNA levels of Twist1 and PDGFB in PAECs derived from IPAH patients or healthy controls (*left*; n = 4–6, *p < 0.05). Graph showing the protein levels of PDGFB in PAECs derived from IPAH patients or healthy controls (*right*; n = 4–6, *p < 0.05). Error bars represent s.e.m. (**b**) Graph showing the mRNA levels of Twist1 and PDGFB in PAECs derived from IPAH patients or healthy controls or in combination with treatment with control siRNA or Twist1 siRNA (*left*; n = 4–6, *p < 0.05). Graph showing the protein levels of PDGFB in PAECs derived from IPAH patients or healthy controls or in combination with treatment with control siRNA or Twist1 siRNA (*right*; n = 4–6, *p < 0.05). Error bars represent s.e.m.

The expression of Twist1 is upregulated in the lungs of patients with PAH^15,16^ and knockdown of endothelial Twist1 prevents hypoxia-induced accumulation of αSMA-positive cells to distal PAs in the mouse model^20^. The mRNA levels of Twist1 and PDGFB were 2.6- and 2.5- times higher, respectively in IPAH patient-derived PAECs compared to those in control healthy PAECs (Fig. 4a). The protein levels of PDGFB measured by ELISA were also 1.5- times higher in IPAH patient-derived PAECs compared to those in control healthy PAECs (Fig. 4a). Consistent with hypoxia-treated HPAE cells (Fig. 3b), Twist1 knockdown using siRNA transfection inhibited the PDGFB mRNA and protein expression in IPAH patient PAECs (Fig. 4b).

**Figure 5:**
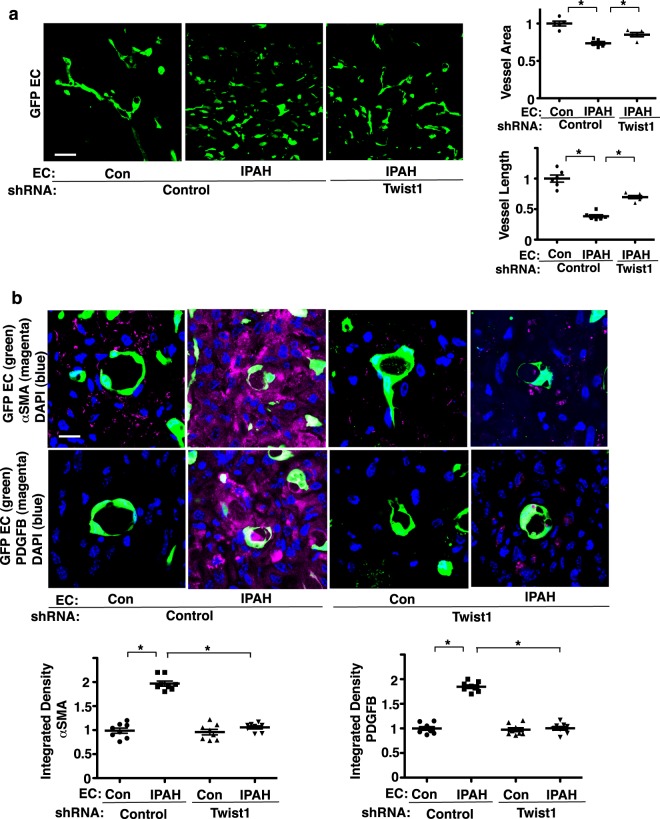
Twist1 mediates accumulation of αSMA-positive cells in the fibrin gel supplemented with IPAH patient ECs and implanted on the mouse lung. (**a**) IF micrographs of fibrin gel supplemented with GFP-labeled PAECs derived from IPAH patients or healthy controls or in combination with treatment with control shRNA or Twist1 shRNA, and implanted on the mouse lung for 7 days showing GFP-labeled blood vessel structures in the fibrin gel. Scale bar, 25 μm. Graphs showing vessel area and length (n = 5–6, *p < 0.05). Error bars represent s.e.m. (**b**) IF micrographs of fibrin gel supplemented with GFP-labeled PAECs derived from IPAH patients or healthy controls or in combination with treatment with control shRNA or Twist1 shRNA, and implanted on the mouse lungs for 7 days showing GFP-labeled blood vessel lumen structure, αSMA expression and distribution, and DAPI (*top*) and GFP-labeled blood vessel lumen structure, PDGFB expression and distribution, and DAPI (*bottom*) in the fibrin gel. Scale bar, 10 μm. Graphs showing integrated fluorescent density to show expression of αSMA and PDGFB (n = 8, *p < 0.05). Error bars represent s.e.m.

Hypoxia induces accumulation of αSMA-positive cells in the gel implanted on the mouse lungs (Fig. 3d–f). Thus, we next examined the IPAH EC-derived blood vessel structure and accumulation of αSMA-positive cells using a mouse lung gel implantation model. When we implanted fibrin gel supplemented with fluorescently labeled IPAH patient lung ECs on the NSG mouse lung (8–10 week old), CD31^+^ blood vessel formation (Supplementary Fig. 3d), which is well developed in the implanted gel supplemented with healthy control PAECs, was attenuated in the gel supplemented with IPAH patient-derived PAECs; vascular density and blood vessel length were lower by 28% and 61%, respectively in the gel supplemented with IPAH patient-derived PAECs, while Twist1 knockdown using shRNA treatment inhibited these effects and partially restored vessel structures in the gel (Fig. 5a). Supplemented IPAH patient PAECs also increased accumulation of αSMA-positive cells in the gel by 1.9-times and stimulated PDGFB expression in the gel by 1.8-times compared to those in the gel supplemented with control healthy PAECs (Fig. 5b). Twist1 knockdown in IPAH patient PAECs suppressed PDGFB expression and accumulation of αSMA-positive cells in the gel (Fig. 5b), suggesting that accumulation of αSMA-positive cells in the gel is inhibited in IPAH-patient derived PAECs by suppressing Twist 1-PDGFB signaling.

## Discussion

**Figure 6:**
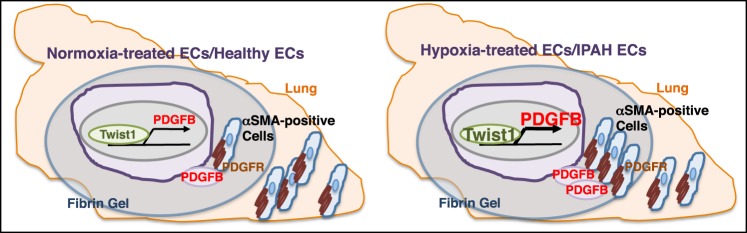
Schematic illustration of accumulation of αSMA-positive cells through Twist1-PDGFB signaling. Schematic illustration demonstrating that endothelial Twist1 stimulates PDGFB expression and mediates hypoxia-induced αSMA-positive cell accumulation in the gel implanted on the mouse lungs. Endothelial Twist1 also mediates accumulation of αSMA-positive cells in the gel supplemented with human IPAH patient-derived ECs and implanted on the mouse lungs. PDGFR: PDGF receptor.

Here we have demonstrated that knockdown of endothelial Twist1 prevents hypoxia-induced DNA synthesis and migration of αSMA-positive cells by decreasing PDGFB expression (Fig. 6). Twist1 overexpression increases the expression of PDGFB in ECs, and CM collected from Twist1 overexpressing ECs induces SMC migration and DNA synthesis *in vitro*. Hypoxia stimulates accumulation of αSMA–positive cells in the gel supplemented with mouse and human ECs and implanted on the mouse lung and upregulates expression of PDGFB in the gel, while Twist1 knockdown in ECs attenuates the effects. The levels of Twist1 and PDGFB are higher in IPAH patient-derived PAE cells, which stimulates accumulation of αSMA–positive cells in the implanted gel, while Twist1 knockdown inhibits the effects. Endothelial Twist 1-PDGFB signaling could be one of the key signaling mechanisms in the pathogenesis of PH.

Our results reveal that CM from ECs overexpressing Twist1 or treated with hypoxia stimulates HPASMC migration and DNA synthesis by increasing PDGFB expression, while knockdown of Twist1 inhibits the hypoxia-induced stimulation of HPASMC DNA synthesis (Figs. 2 and 3). Twist1 is a bHLH transcription factor and controls expression of other angiogenic genes which contain an E-box in their promoter region (e.g.,TGFβ2^28^, VEGFR2^9^, Tie2^13^, TGFβR2^20^)^30^, and may regulate endothelial and PASMC behaviors in a cooperative way. In addition to PDGFB, PDGFA is also involved in PH pathology^32^. Other pathways known to mediate the PH pathology (e.g., eNOS^42^, High Mobility Group AT-hook 1 (HMGA1)^22^, smad^20,43^, PGC1α/TFAM^44^) may be involved in the mechanism. For example, Twist1 controls PGC1α expression in brown fat tissue and regulates cellular metabolism^45^, which also plays important roles in PH pathology^44^. Twist1 also interacts with other signaling molecules (e.g., Wnt^25^), which may contribute to hypoxic PH. Thus, although hypoxia increases Twist1 expression, the effects of Twist1 overexpression may be different from those of hypoxia, which also stimulates other pathways. We have reported that Twist1 Ser42 phosphorylation plays a key role in PH pathology in the hypoxia-induced mouse PH model^20^. Since Twist1S42A mutant construct decreases PDGFB expression in HPAE cells (not shown), Twist1 Ser42 phosphorylation may contribute to PH pathology by changing PDGFB expression as well. In fact, the hypoxia-induced changes in the levels of Twist1 are modest compared to those in PDGFB levels, suggesting the effects of post-translational modification of Twist1 (e.g., Twist1 Ser42 phosphorylation^20^) on PDGFB expression. Factors other than Twist1 may also be involved. It is reported that HIF1α controls PDGFB expression in breast cancer cells^46^. Since HIF1α controls a number of downstream signaling pathways, these other pathways could be involved in the hypoxia-induced change in PDGFB expression. Nevertheless, given that Twist1 knockdown decreased the hypoxia-induced PDGFB levels to the baseline levels (Fig. 3b), Twist1 may be one of the major molecules contributing to this mechanism.

We found that when we implanted fibrin gel supplemented with ECs isolated from *B6-GFP* mouse lungs to *B6-mRFP* mouse lungs and treated the mice with hypoxia, αSMA-stained mRFP-positive cells accumulated in the gel (Supplementary Fig. 3b). These results suggest that αSMA-positive cells in the gel seem to be recruited from host mouse lungs. The origin of these recruited αSMA-positive cells remains unclear. Given that the sub-plural area of the mouse lung, where the gel is implanted, is generally devoid of muscularized vessels, these αSMA-expressing cells may be myofibroblasts derived from fibroblasts in the host lungs^47^, EndMT-derived αSMA-positive cells from the host lung ECs^20^, or progenitor cells which differentiate into αSMA-positive cells^2,48,49^. Knockdown of endothelial Twist1 attenuates hypoxia-induced SMC DNA synthesis and migration *in vitro* and accumulation of αSMA-positive cells in the gel implanted on the mouse lungs through PDGFB signaling (Fig. 3). αSMA-positive cells (*e.g*., pericytes, myofibroblasts) and other lung cells (*e.g*., epithelial cells, immune cells)^50^ recruited into the gel may reciprocally interact and/or secrete angiogenic and other chemical factors, which results in altering Twist1-PDGFB signaling and indirectly affects vascular structures and αSMA-positive cell recruitment in a spatiotemporal manner. Hypoxia-induced accumulation of αSMA-positive cells may not be mediated through endothelial Twist1-PDGFB signaling but through hypoxic effects on αSMA-positive cells or other lung cells. Supplementation of differently labeled other lung cells will enable us to further understand the mechanism by which endothelial Twist1 controls vascular remodeling in the gel.

In addition to angiocrine signaling such as PDGFB, hypoxia also induces vascular structural changes through other mechanisms. For example, hypoxia induces an inflammatory response in the lung, which is critical for the later development of hypoxic PH^51,52^. Twist1 is involved in various inflammatory pathways^13,53–55^. PDGFB is also involved in the inflammatory pathways in the lung and contributes to airway remodeling in asthma^56^ and pulmonary fibrosis^57^. Thus, inhibition of Twist1 and/or PDGFB expression may attenuate hypoxic PH by inhibiting the inflammatory response to hypoxia as well. EndMT is one of the important processes of the PH pathology and may change the behaviors of surrounding cells and microenvironment (e.g., shear flow, vascular mechanics), which leads to the subsequent vascular structural changes. We have reported that EndMT and accumulation of αSMA-positive cells in the gel are stimulated by treatment with hypoxia for 3 days through Twist1 signaling^20^ (Fig. 3). Twist1 interacts with other EMT/EndMT genes (e.g., Snail/Slug^20,22^) and may contribute to hypoxic PH. Although EndMT was stimulated by treatment with hypoxia for 3 days through Twist1 signaling^20^ (Fig. 3), EndMT was not clearly detected in ECs derived from IPAH patients in the gel (Fig. 5b). EndMT may ensue in only a small percentage of IPAH patient-derived ECs or during the specific time point. Precise time course IHC analysis in combination with supplementation of inflammatory cells in the gel or manipulation of expression of inflammatory mediators or EndMT-related genes will clarify the mechanism.

In addition to hypoxic PH^20^, we have found that the levels of Twist1 increase in IPAH patient ECs. The mechanism other than hypoxia may be involved in the upregulation of Twist1 in IPAH patient ECs. It is reported that the levels of the chromatin-associated transcriptional regulator, HMGA1, increase in IPAH ECs, which induces EndMT in pulmonary hypertension; knockdown of HMGA1 inhibits EndMT gene expression induced by loss of BMPR2^22^. Since Twist1 is involved in EndMT, HMGA1 may control Twist1 expression in IPAH patient ECs. Other signaling pathways contributing to PH pathology, including TGFβ-smad signaling^20,58^, TNFα-NFkB signaling^59,60^, IL6^61,62^, and CD44-LOX signaling^63,64^ are known to induce Twist1 expression in cancer cells and may be involved in the mechanism.

We have investigated the role of Twist 1-PDGFB signaling in αSMA-positive cell accumulation using ECs isolated from IPAH patient PA with a variety of conditions that can affect endothelial signaling and angiogenic activity. We excluded the samples from >50 years old patients, which are more susceptible to COPD or pulmonary fibrosis that affect other mechanisms, and collected EC samples from the region >5 mm in diameter. However, the heterogeneity of the samples due to cardiopulmonary condition (e.g., chronic lung diseases, inflammation), obesity, sex, and type-2 bone morphogenetic protein receptor (BMPR2) mutations may impact blood vessel formation, SMC DNA synthesis and migration and the signaling pathways^2^. It is well known that BMPR2 mutations contribute to severity of PH phenotype for vascular remodeling (SMC accumulation, EndMT)^2,17^. However, due to sample availability, we did not investigate the effects of BMPR2 mutation in human IPAH patient samples in this study. Further investigation in another cohort with a larger sample size will be necessary to elucidate the mechanism of Twist1-PDGFB signaling in the PH pathology.

In summary, we have demonstrated that endothelial Twist1 controls PDGFB expression and mediates hypoxia-induced DNA synthesis of SMCs *in vitro* and accumulation of αSMA-positive cells in the gel implanted on the mouse lungs. Twist1 knockdown in IPAH patient-derived PAECs attenuates accumulation of αSMA-positive cells in a gel implanted on the mouse lung. These findings suggest that Twist1-PDGFB signaling may be one of the central pathways involved in the pathogenesis of PH.

## Supplementary information

Supplementary Information.
