# Supplementary material for: Endothelial Twist1-PDGFB signaling mediates hypoxia-induced proliferation and migration of αSMA-positive cells
Source: Sci Rep. 2020 May 5;10:7563. doi: 10.1038/s41598-020-64298-5 (PMC7200682; doi:10.1038/s41598-020-64298-5)
Supplement: Supplementary file 1 — Supplementary Information. [file 41598_2020_64298_MOESM1_ESM.pdf]

Endothelial Twist1-PDGFB signaling mediates hypoxia-induced proliferation and migration of αSMA-positive cells

Akiko Mammoto[1,2]*, Kathryn Hendee[1], Megan Muyleart[1], and Tadanori Mammoto[1]*

[1]Department of Pediatrics and [2]Department of Cell Biology, Neurobiology and Anatomy, Medical College of Wisconsin, Milwaukee, WI 53226.

*To whom correspondence should be addressed: Akiko Mammoto, MD, PhD and Tadanori Mammoto, MD, PhD. Medical College of Wisconsin, 8701 Watertown Plank Rd, Milwaukee, WI 53226, USA (TEL: 1-414-955-3612; FAX: 1-414-266-6979; e-mail: amammoto@mcw.edu tmammoto@mcw.edu)

*These authors are equally contributed to the manuscript.

Supplementary Figure Legends:

Supplementary Fig. 1. The effects of knockdown or overexpression of Twist1 on Twist1 expression in HPAE cells. Original immunoblots for the images in Fig. 1a showing Twist1 and β-actin protein levels in HPAE cells treated with lentivirus overexpressing Twist1 or control virus, or Twist1 siRNA or control siRNA with irrelevant sequences.

Supplementary Fig. 2. Flow cytometry analysis of ECs isolated from mouse lungs and DNA synthesis. a) Flow cytometry analysis of pulmonary ECs isolated from mouse lungs. **b)** Flow cytometry analysis of BrdU-positive HPASMCs treated with/without PDGFB. Graph showing % of BrdU-positive HPASMCs treated with/without PDGFB (n=3, *, $p<0.05$). Error bars represent s.e.m.

Supplementary Fig. 3. Hypoxia induces accumulation of αSMA-positive cells in the gel implanted on the mouse lung. a) IF micrograph of fibrin gel implanted on the C57BL6 mouse lung for 7 days stained with 2nd antibody alone. Scale bar, 50 μm. **b)** IF micrograph of fibrin gel supplemented with GFP-labeled *B6-GFP* mouse lung ECs implanted on *B6-mRFP* mouse lung for 7 days and treated with hypoxia for the last 3 days; GFP-labeled blood vessel lumen structure, mRFP-labeled host mouse lung cell distribution, and αSMA expression and distribution in the fibrin gel. Scale bar, 10 μm. **c)** IF micrographs of fibrin gel supplemented with GFP-labeled HPAE cells implanted on NSG mouse lung for 7 days and treated with hypoxia for the last 3 days; GFP-labeled

blood vessel structure, αSMA expression and distribution, and DAPI in the fibrin gel. Scale bar, 100 μm. **d)** IF micrographs of fibrin gel supplemented with GFP-labeled human PAECs, and implanted on the mouse lung for 7 days showing GFP-labeled blood vessel structure and CD31 expression in the fibrin gel. Scale bar, 50 μm.

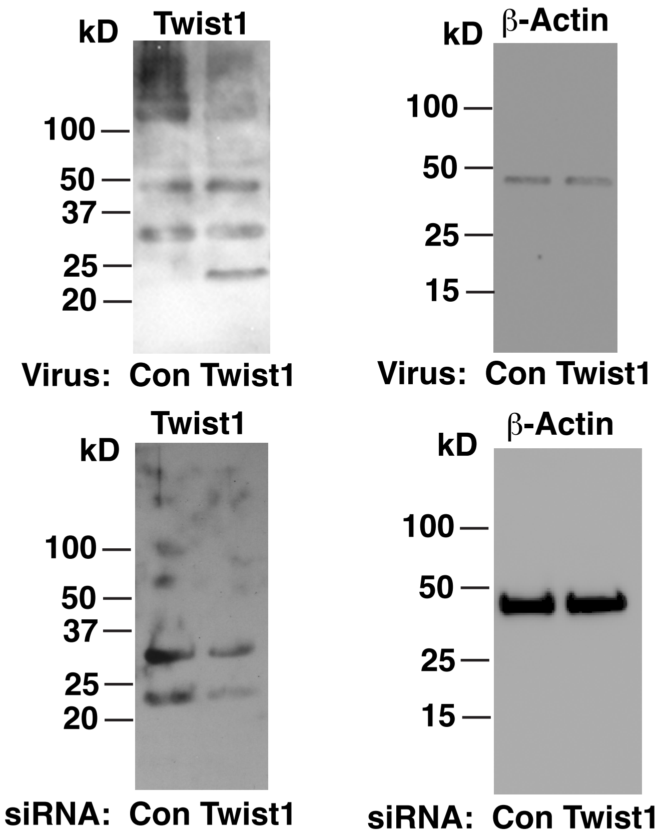

Supplementary Fig. 1

a

b

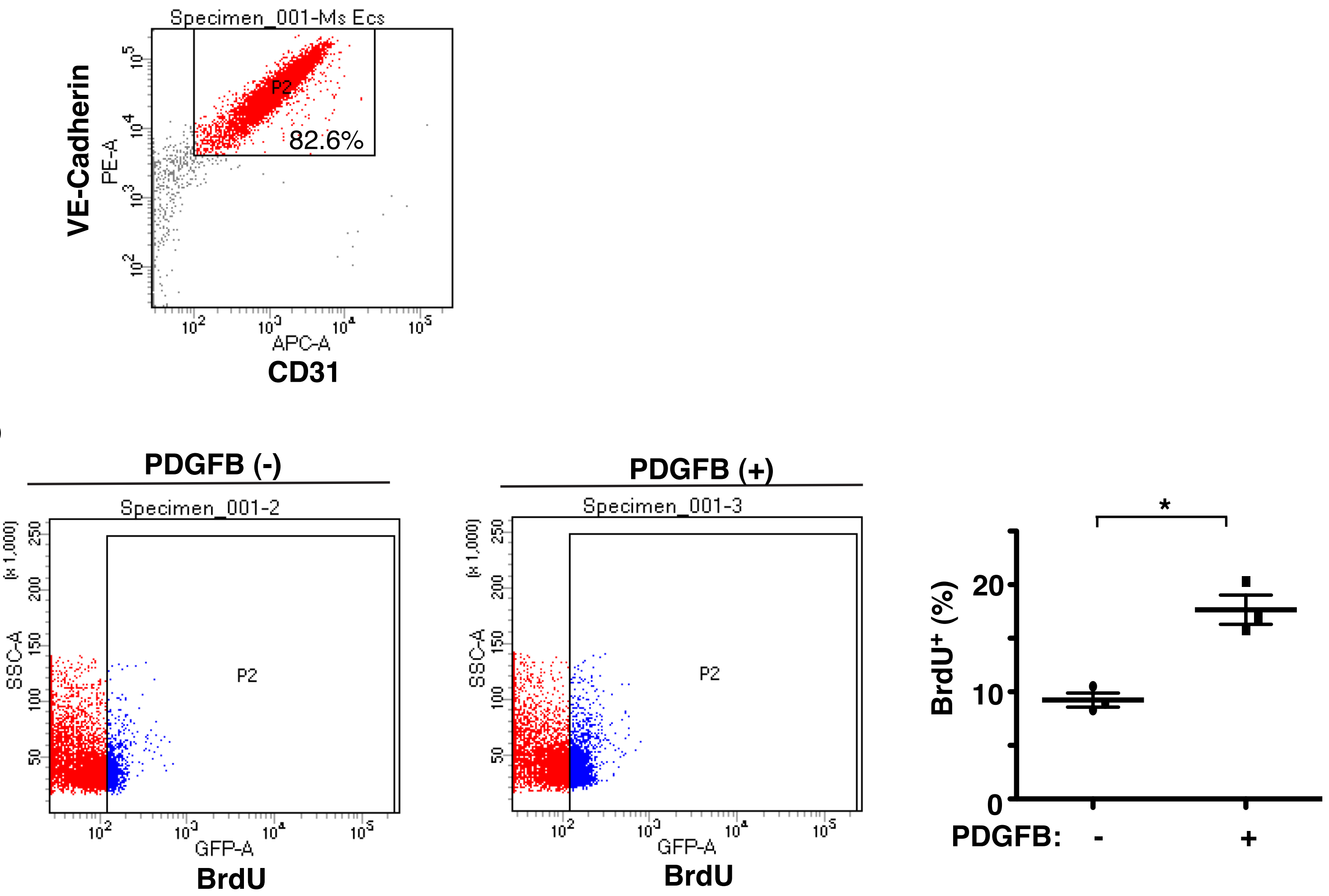

Supplementary Fig. 2

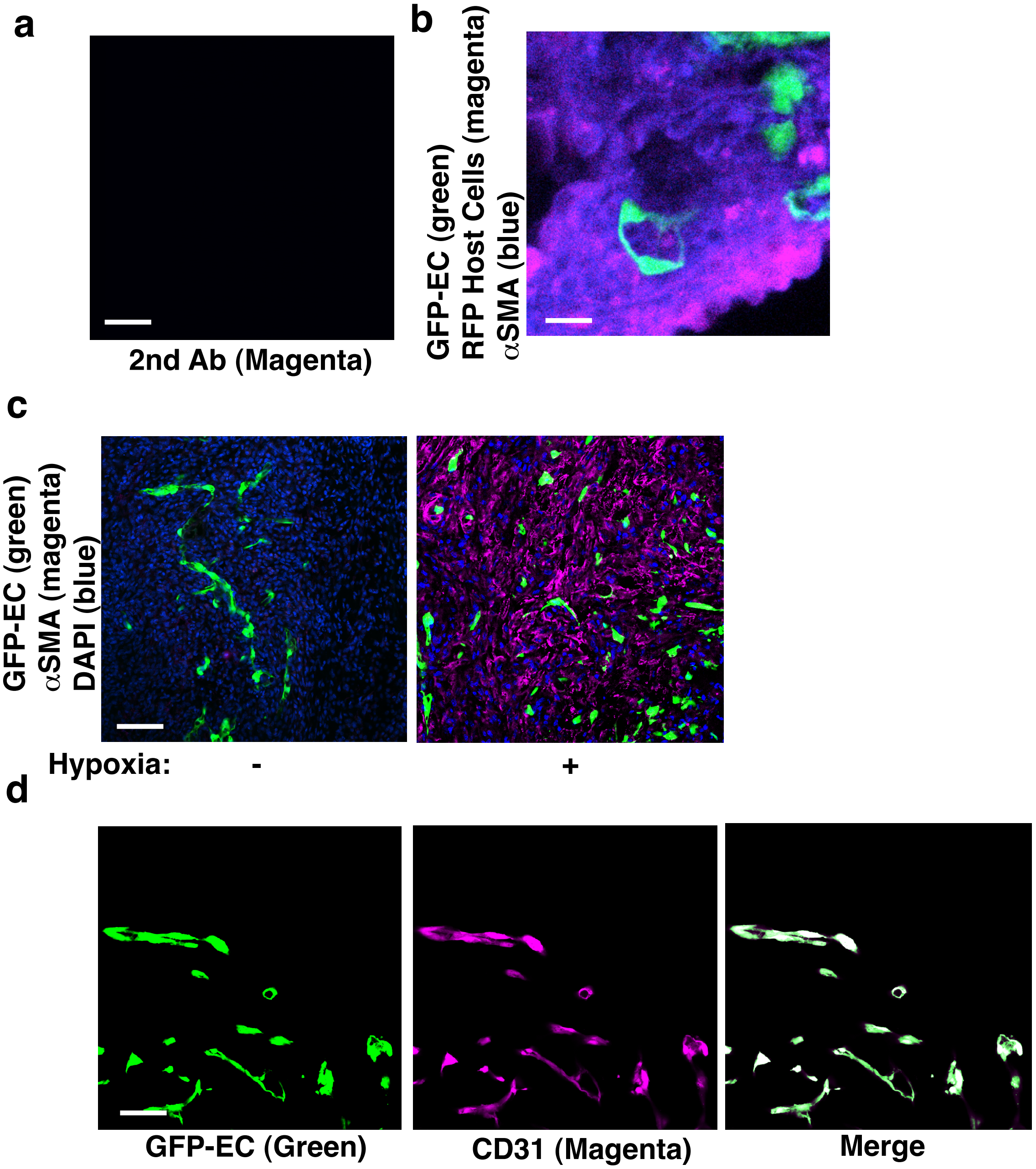

Supplementary Fig. 3
